# EEG electrode setup optimization using feature extraction techniques for neonatal sleep state classification

**DOI:** 10.3389/fncom.2025.1506869

**Published:** 2025-01-31

**Authors:** Hafza Ayesha Siddiqa, Muhammad Farrukh Qureshi, Arsalan Khurshid, Yan Xu, Laishuan Wang, Saadullah Farooq Abbasi, Chen Chen, Wei Chen

**Affiliations:** ^1^Center for Intelligent Medical Electronics, Department of Electronic Engineering, School of Information Science and Technology, Fudan University, Shanghai, China; ^2^Department of Electrical Engineering, Namal University Mianwali, Mianwali, Pakistan; ^3^Department of Electrical Engineering, Engineering Institute of Technology, Melbourne, VIC, Australia; ^4^Department of Neurology, Children's Hospital of Fudan University, National Children's Medical-Center, Shanghai, China; ^5^Department of Neonatology, Children's Hospital of Fudan University, Shanghai, China; ^6^Department of Electronic, Electrical and Systems Engineering, University of Birmingham, Birmingham, United Kingdom; ^7^Human Phenome Institute, Fudan University, Shanghai, China; ^8^School of Biomedical Engineering, The University of Sydney, Sydney, NSW, Australia

**Keywords:** EEG, sleep analysis, neonatal sleep state classification, principal component analysis, SMOTE, LSTM

## Abstract

An optimal arrangement of electrodes during data collection is essential for gaining a deeper understanding of neonatal sleep and assessing cognitive health in order to reduce technical complexity and reduce skin irritation risks. Using electroencephalography (EEG) data, a long-short-term memory (LSTM) classifier categorizes neonatal sleep states. An 16,803 30-second segment was collected from 64 infants between 36 and 43 weeks of age at Fudan University Children's Hospital to train and test the proposed model. To enhance the performance of an LSTM-based classification model, 94 linear and nonlinear features in the time and frequency domains with three novel features (Detrended Fluctuation Analysis (DFA), Lyapunov exponent, and multiscale fluctuation entropy) are extracted. An imbalance between classes is solved using the SMOTE technique. In addition, the most significant features are identified and prioritized using principal component analysis (PCA). In comparison to other single channels, the C3 channel has an accuracy value of 80.75% ± 0.82%, with a kappa value of 0.76. Classification accuracy for four left-side electrodes is higher (82.71% ± 0.88%) than for four right-side electrodes (81.14% ± 0.77%), while kappa values are respectively 0.78 and 0.76. Study results suggest that specific EEG channels play an important role in determining sleep stage classification, as well as suggesting optimal electrode configuration. Moreover, this research can be used to improve neonatal care by monitoring sleep, which can allow early detection of sleep disorders. As a result, this study captures information effectively using a single channel, reducing computing load and maintaining performance at the same time. With the incorporation of time and frequency-domain linear and nonlinear features into sleep staging, newborn sleep dynamics and irregularities can be better understood.

## 1 Introduction

Sleep is a natural, repetitive period of rest and unconsciousness that is required for the healthy functioning of both the body and the mind (Baker, 1985). During sleep, the body undergoes a series of stages, and each stage offers a distinct benefit and influences numerous physiological and psychological functions, including memory consolidation, cognitive function, mood regulation, and physical ability restoration (Song et al., 2024). As a general rule, sleep involves a reduction in consciousness and awareness of the environment, a reduction in voluntary muscle contraction, a decrease in metabolism, and a reversible and periodic state (Arif et al., 2021). As a result of inadequate sleep, cognitive function can be impaired, the immune system weakens, and the risk of chronic diseases increases. These diseases, including obesity, diabetes, heart disease, and hypertension can increase (Khan S. et al., 2020; Killick et al., 2022; Parish, 2009; Pan et al., 2024; Chen and Zhu, 2024). The recommended amount of sleep for adults is between 7–9 h per night (Baker, 1985). Neonates, however, have shorter sleep cycles, making them more susceptible to unpredictable sleep patterns. It is common for infants to sleep approximately 16–17 h per day, but the duration varies depending on the individual.

Just like adults, neonates also go through various sleep stages (Newson, 2017). In neonates, there are two main stages of sleep: Active Sleep (AS) and Quiet Sleep (QS). The infant is in AS state when he or she has rapid eye movements, involuntary breathing, and a rapid heart rate. During this state of sleep, babies are able to move, express their facial expressions, and are even capable of sucking. The development of the brain and the learning process of the infant are directly related to AS. During QS, babies' hearts beat slower, their breathing is regular, and they do not move very much. Physical development and growth are strongly influenced by QS. In addition to the AS and QS stages, infants also experience a third transitional stage in their sleep cycle, which combines both the AS and QS stages. There are two main differences between Active Sleep 1 (AS1) and Active Sleep 2 (AS2). The main difference is how much the brain is active and how much the eyes move. In AS1, highly irregular brain waves and frequent changes in the eye movements are characterized, however, in AS2, the eye movements are less frequent and the brain activity is more regular. As an alternative, QS can be divided into two categories, one of which is Quiet Sleep 1 and the other is Quiet Sleep 2. There is a significant difference between QS1 and QS2, as the movements and brain waves differ significantly. In QS1, there is increased activity, with abnormal brain activity and body movements. As opposed to this, QS2 is a quieter state in which the brain is more active regularly and the body is less active.

### 1.1 Main motivation of the proposed approach

The primary objective of this study is to evaluate the potential for differentiating neonatal sleep into five states using single-channel and multi-channel EEG data. To identify the best electrode configuration and minimize technical difficulties and potential irritation of the skin that may occur during the collection of EEG data for neonates, data collected from single-channel EEG is being used. The LSTM algorithm is used to classify an infant's sleep into five stages by using various EEG features including three novel features (Detrended Fluctuation Analysis (DFA), Lyapunov exponent, and multiscale fluctuation entropy).

### 1.2 Main contributions

There are five main parts to this study, and they are outlined below:

1. Extraction of multiple linear and non-linear features in the time and frequency domains.
2. As a non-linear state-of-the-art approach for EEG-based neonatal sleep staging, Detrended Fluctuation Analysis (DFA), Multiscale Fluctuation Entropy (MFE), and Lyapunov exponent are taken into account.
3. To address class imbalance, the SMOTE technique is used to balance the dataset.
4. PCA-based feature normalization and selection.
5. Using both one channel at a time as well as different combinations of multiple channels at the same time to classify five different sleep states.
  In addition, the study examines the optimal configuration of EEG electrodes for five-state classification, including how many electrodes to use and where they should be placed. To reduce complexity, skin irritation risk, and cost in neonatal sleep studies, this study evaluated sleep stage classification accuracy using various electrode setups.

This article is structured as follows: Section 2 reviews relevant literature; Section 3 presents the methodology that has been proposed and its findings based on the proposed methodology; and A discussion of the proposed work's findings and limitations is provided in Section 4. In Section 5, the proposed study's conclusions are presented.

## 2 Related work

Human sleep behavior was first studied using electroencephalography (EEG) in Loomis et al. (1937). With the advent of deep and machine learning algorithms, there are a number of algorithms that have been developed in order to categorize adult sleep patterns (Lajnef et al., 2015; Xiao et al., 2013; Fonseca et al., 2016; Gudmundsson et al., 2005; Turnbull et al., 2001; De Wel et al., 2017; Dereymaeker et al., 2017; Koolen et al., 2017; Pillay et al., 2018; Ansari et al., 2020; Fraiwan and Lweesy, 2017). Pillay et al. (2018) developed a model based on multichannel EEG recordings to automatically classify a person's sleep using Hidden Markov Models (HMMs) and Gaussian Mixture Models (GMMs) and the Cohen's Kappa of the model was 0.62, which was higher than the Cohen's Kappa of a GMMs. A CNN was also used to classify sleep stages 2 and 4 (Ansari et al., 2020). Wake states were not included in these techniques. In Awais et al. (2020), developed using pre-trained CNNs to extract features to classify neonatal sleep and wake. According to this study, a model that has been pre-trained was inadequate for categorizing sleep and wake in neonates with high accuracy. In Awais et al. (2021), the authors combine deep convolutional neural networks (DCNN) with self-learning models to classify infant sleep and waking states based on video facial expressions. EEG video data could be classified accurately at 93.8 ± 2.2% and F1-scores were 0.93 ± 0.3. It is worth mentioning that video EEG data can contain infant's faces and voices, creating privacy issues as a result.

A study conducted in 2021 by authors in Lee et al. (2021) with IR-UWB radar to classify non-contact sleep and wake in infants found an accuracy of 75.2%. According to another study that classified quiet sleep based on EEG data, the value of Kappa was 0.77 ± 0.01 for eight-channels and 0.75 ± 0.01 for single bipolar-channel (Ansari et al., 2021). According to a study conducted by Abbasi et al., a MLP neural network algorithm developed for binary classification of neonatal sleep has been tested and the value of Kappa has been determined to be 62.5%, and the accuracy has been determined to be 82.5% using the algorithm (Abbasi et al., 2020). A three-state classification of the same dataset was performed in 2022 using bagging and stacking ensemble methods with an accuracy of 81.99% (Abbasi et al., 2022). By using publicly available single-channel EEG datasets, Yu et al. (2022) classified neonate's sleep patterns into W, N1, N2, and N3. The multi-resolution attention sleep network (MRASleepNet) module was tested to classify sleep patterns. A feature extraction module, a multi-resolution analysis module, and a gated MLP module were all included in the algorithm. Through an adaptive boosting (AdaBoost) classifier, Arasteh et al. (2023) classified AS and QS with 81% accuracy achieved through cross-validation of tenfold. The AutoML-based Random Forest estimator obtained an accuracy rate of 84.78% and a kappa rate of 69.63% for prediction of neonatal sleep and wake states in Siddiqa et al. (2023). According to Ansari et al. (2018), an 18-layer CNN is used to detect neonatal QS sleep stages with multichannel EEG data. A Multi-Scale Hierarchical Neural Network (MS-HNN) has been developed in Zhu et al. (2023) Using two, four, and eight channels to automatically classify neonatal sleep states. Features including temporal information were extracted using multi-scale convolutional neural networks (MSCNN). They attained an accuracy of 75.4% using single-channel classification and 76.5% using a combination of eight channels for three-stage classification. Supratak et al. (2017) performed classification of sleep states in newborn with DeepSleepNet and attained 69.8% accuracy. In Eldele et al. (2021), authors proposes AttenSleep, a deep learning approach based on attention for sleep stage classification. Instead of using RNNs, AttenSleep uses multi-head attention (MHA) to identify the chronological relationship among different stages of neonatal sleep. Using multi-branch CNN and reached classification accuracy of 74.27% with single channel and 75.36% with four channel EEG, Hafza et al. proposed three-state EEG-based neonatal sleep state classification (Siddiqa et al., 2024). The authors incorporated 74 features in the time and frequency domains.

As a result of limited classifications, privacy concerns, long training times, and poor accuracy, existing approaches for recognizing infant sleep stages have significant limitations. Without taking into account awake, it is challenging to classify newborn sleep accurately. Non-linear features which aren't typically included in current sleep staging methodologies for neonates include DFA, MFE, and the Lyapunov Exponent. Further, these methods require multichannel EEG data, which disrupts the skin and causes discomfort, highlighting the need for methods that are non-invasive. To effectively differentiate between the five-state sleep patterns in newborns, it is crucial to develop a dependable and privacy-conscious strategy that ensures high accuracy while minimizing any potential negative consequences.

## 3 Materials and methods

**Figure 1:**
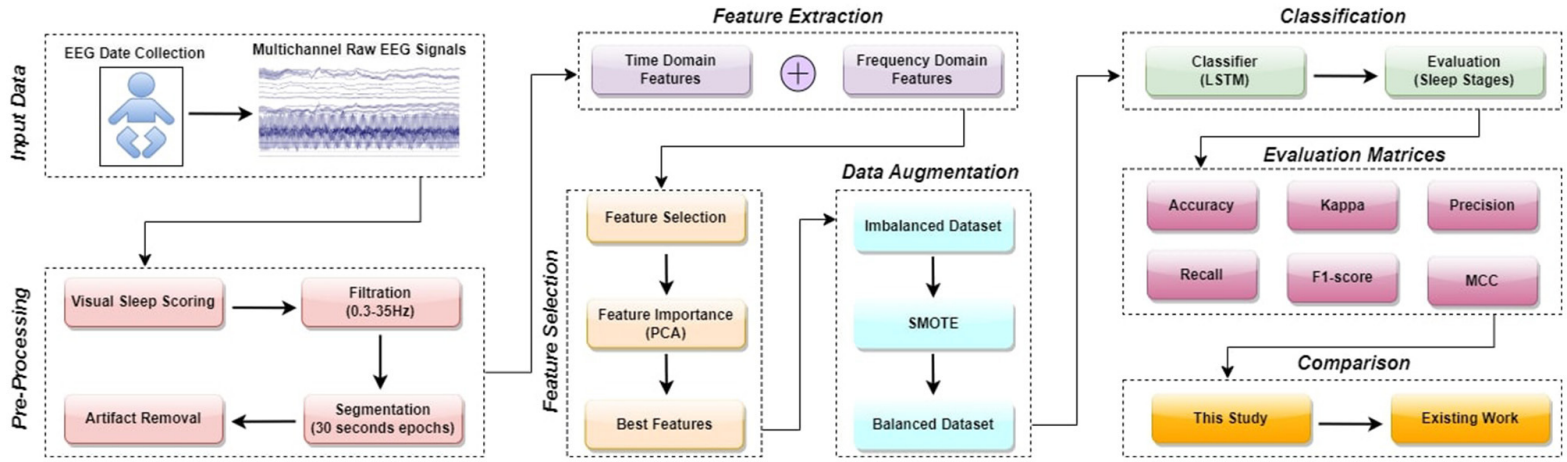
A detailed flowchart of the proposed methodology.

An LSTM model for the categorization of neonate's sleep into five distinct states is introduced in this article. In this section, a step-wise overview of the proposed design is provided. The sequential flowchart of the proposed methodology is illustrated in Figure 1. The process can be further explained by following these steps:

### 3.1 EEG dataset

**Figure 2:**
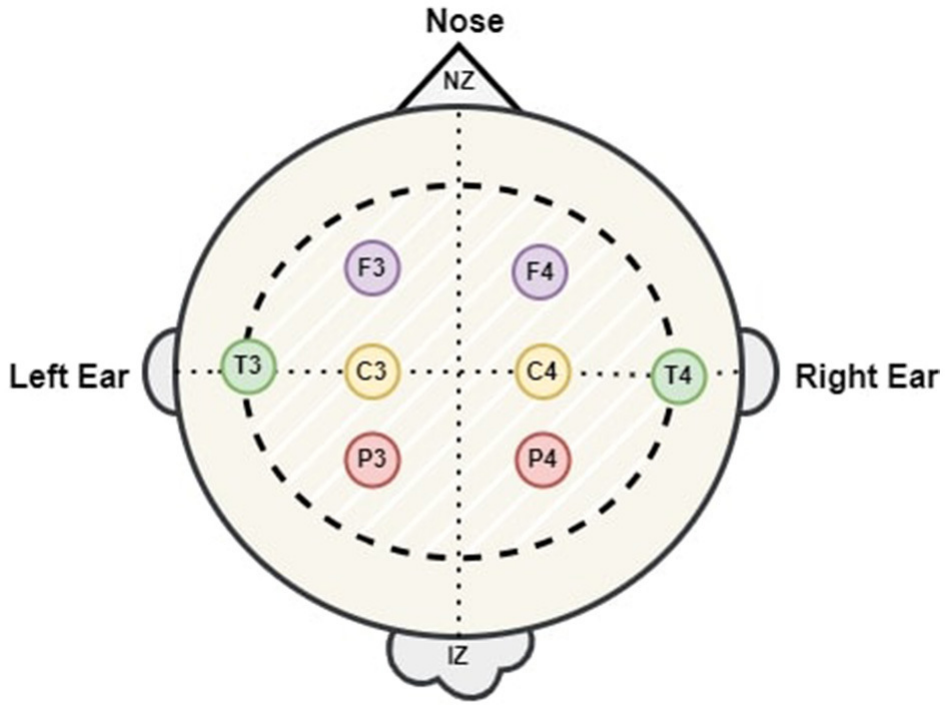
The positioning of the 8 electrodes utilized in this research.

EEG data was obtained from 64 neonates admitted to the neonatal intensive care unit (NICU) at Children's Hospital of Fudan University (CHFU), located in China. This work has obtained approval from the Research Ethics Committee of Children's Hospital of Fudan University, with the assigned Approval No. (2017) 89. The proposed model was tested and trained using these EEG recordings. The data was collected during observations of neonates at various time points. A full 10-20 electrode installation system comprises the following 17 electrodes: “FP1,” “FP2,” “F3,” “F4,” “F7,” “F8,” “C3,” “C4,” “P3,” “P4,” “T3,” “T4,” “T5,” “T6,” “O1,” “O2,” and “Cz.” Every letter is associated with a distinct region or lobe of the brain. The letters FP, F, T, P, O, and C represent the prefrontal, frontal, temporal, parietal, occipital, and central regions of the brain. Throughout this time frame, we have witnessed a multitude of sleep patterns. The study included EEG recordings from eight specific channels: “C3,” “C4,” “F3,” “F4,” “P3,” “P4,” “T3,” and “T4.” The NicoletOne multi-channel EEG equipment was utilized for the purpose of recording of the EEG data at a sampling rate of 500 Hz. The NicoletOne EEG devices have lightweight electrode caps that securely fasten scalp electrodes, ensuring accurate signal capture. The NicoletOne EEG device enables the acquisition of high-quality EEG signals with a high sampling rate of up to 2 kHz and a broad frequency range spanning from 0.053 to 500 Hz. Figure 2 illustrates the locations of the eight electrodes used in this study, in accordance with the 10–20 system recommendations. Nz represents the foundation of the nose, whereas Iz indicates the protuberance.

### 3.2 Visual sleep scoring of EEG dataset

The EEG segments were visually classified by experienced neurologists from Fudan children hospital Shanghai, based on five main categories: Wakefulness, AS1, AS2, QS1, and QS2. When classifying sleep states, non-cognitive features were employed in conjunction with the EEG. In addition, the experts took into account NICU videos when conducting the annotating procedure. Table 1 provides comprehensive details regarding the dataset (Siddiqa et al., 2024).

**Table 1:** A detailed description of the dataset (Siddiqa et al., 2024).

**Variable/category**	**Descriptions**
Sampling frequency	500 Hz
Number of channels	8
Number of subjects	64
Number of epochs	16,803
Gestational age	38.3 ± 1.8 (wk+d)
Post-menstrual age	40.5 ± 1.7 (wk+d)
Gender	32 males and 32 females
Sleep time	1.44 ± 0.57 h
Wake time	0.71 ± 0.57 h
Weight	3.3 ± 0.6 kg
Reason for admittance	Septicemia, Hyperbilirubinemia, and etc.

### 3.3 EEG dataset pre-processing

Distortion and artifacts during recording have an impact on the quality and reliability of the EEG data. The EEG data was recorded at a sampling rate of 500 Hz. These EEG recordings underwent a pre-processing phase to eliminate noise and artifacts. The pre-processing involves the following steps:

1. An FIR (Finite Impulse Response) filter was employed to eliminate undesired signals from EEG recordings within the frequency range of 0.3 to 35 Hz (High Pass = 0.3 Hz and Low Pass = 35 Hz).
2. The EEG signals that have been processed by a filter are now divided into segments of 30 seconds each.
3. Following the process of segmentation, a label given by experienced neurologists is issued to each epoch. The five-state classification assigns W as the first state, AS1 as the second state, QS1 as the third state, QS2 as the fourth state, and AS2 as the fifth state.
4. Artifacts and noise were introduced into the EEG recordings during the recording and processing stages. Consequently, following the pre-processing stage, there are a total of 16,803 epochs available for the testing and training over the channels “C3,” “C4,” “F3,” “F4,” “P3,” “P4,” “T3,” and “T4.”

### 3.4 Feature extraction

The extraction of features from the EEG signals is essential for categorization. Since it aids in distinguishing among various sleep stages or events by analyzing patterns and characteristics. Interpreting EEG data can be difficult because of the fact that there are so many signals that change over time produced as a result of electrical activity in the brain. This work utilizes linear and non-linear feature extraction techniques to decrease the number of dimensions of the data that need to be analyzed and identify relevant characteristics of the data that can be employed for categorization purposes, such as frequency and time distributions (Gosala et al., 2023; Khan J. S. et al., 2020). Overall, 94 linear and non-linear features were retrieved from each channel utilizing various procedures, which include:

#### 3.4.1 Time domain features

- *Statistical features of the EEG signal and its first and second derivatives:* To study and summarize the main statistical features of the EEG signal as well as its derivatives, it would be helpful to pull out features in the time domain of the dataset in order to group newborn's sleep stages (Siddiqa et al., 2023). The extraction of time-domain features is a valuable as well as practical approach to evaluating EEG data, serving both clinical and research applications. Initially, the signal's nine statistical characteristics (mean, median, standard deviation, minimum, maximum, kurtosis, skewness, variance, and range) are computed. Subsequently, an identical collection of five statistics is computed for both the first derivative of the signals obtained from the EEG as well as the second derivative.
- *Detrended fluctuation analysis (DFA):* It is a non-linear feature, computed to measure if EEG signals are correlated at either long or short ranges or if they are self-similar. It also quantifies the extent to which the fluctuations of a signal, after being combined and detrended at various epochs, diverge from a linear pattern (Lal et al., 2023). The DFA, or Detrended Fluctuation Analysis, is a mathematical measure that quantifies the scaling exponent characterizing the connection between the amplitude of fluctuations and the corresponding time scales. The equation for calculating the DFA is as follows:
  (1) $$\begin{array}{l} F\left(n\right)=\sqrt{\frac{\sum {\left[Y\left(i\right)-y\left(i\right)\right]}^{2}}{n}} \end{array}$$
  The fluctuations are represented by F(n) for window size n, the integrated or cumulative profiles of the EEG data are represented by Y(i), and the regression line is represented by y(i). To calculate the DFA, this study uses the nolds.dfa() function from the nolds library. The EEG signal data are converted into NumPy arrays and the DFA is calculated. Conversely, lower values of DFA imply less reliable correlations or less predictable patterns, whereas high values of DFA show better correlations over long distances or similarity to itself, and this implies that a signal is more structured and easier to predict. By utilizing the various DFA characteristics, individuals can acquire a deeper understanding of what is going on within the signal as well as its intricacy. These characteristics have the potential to be advantageous in a range of different applications, such as the evaluation of signals, statistical analysis of time series, and biological studies as well.
- *Lyapunov exponent:* The Lyapunov exponent, a nonlinear feature, measures the responsiveness of a dynamical system to its initial circumstances (Cao et al., 2023). EEG feature extraction is a valuable tool for understanding the predictability and stability of brain processes. The Rosenstein approach is employed to calculate the Lyapunov exponent based on EEG data. The algorithm involves defining parameters for data embedding, initializing tangent vectors, and performing Jacobian matrix calculations. The QR decomposition is used to orthogonalize the tangent vectors, which are then normalized to quantify the system's sensitivity to perturbations. Logarithms of Jacobians divided by tangent vectors and iterations determine the Lyapunov exponent. The Lyapunov exponent (λ) is given by:
  (2) $$\begin{array}{l} \lambda =\frac{1}{N-1}\overset{N-1}{\underset{n=1}{\sum }}x \end{array}$$
  where,
  (3) $$\begin{array}{l} x=\log\left(\frac{d\left(n+1\right)}{d\left(n\right)}\right) \end{array}$$
  This sum is taken over time steps from *n* = 1 to *N*−1, where N is the total number of time steps. The variable *x* describes the relative changes in distances between nearby trajectories in the dynamical system, which is used in calculating the Lyapunov Exponent to characterize the behavior and predictability of the dynamical system. The term *x* represents the logarithm between *d*(*n*+1) and *d*(*n*), which are the distances of the perturbed trajectory at time n+1 and n, respectively. The Lyapunov exponent values not only offer insight into the classification of sleep stages in EEG analysis but also provide information about how complex neonatal sleep dynamics are and the extent to which they can be predicted.
- *Multiscale fluctuation entropy (MFE):* Within the scope of the present study, MFE values have been computed for every epoch of EEG data in order to measure the degree of complexity as well as the irregularity of the signal (Wan et al., 2023). The standard deviation is calculated segment by segment using a scaling factor. The procedure entails multiple sequential stages. The EEG signal is divided into segments according to the scale factor. The variation of each segment is determined by comparing the standard deviation of each segment to the mean of each segment and then calculating the average of the standard deviations of each segment. There is a formula known as the Shannon entropy formula, which is employed to calculate entropy for an ensuing string of fluctuations. Mathematically, MFE can be written as:
  (4) $$\begin{array}{l} MFE=\frac{1}{K}\overset{K}{\underset{k=1}{\sum }}H_{k} \end{array}$$
  In this case, Shannon entropy at each scale is represented by *H*_*k*_, and total number of scales is represented by *K*. The objective of this work is to obtain a deeper understanding of the complexities and inconsistencies of neural activity at different levels by calculating MFE values. It specifically aids in the study of EEG data, which offers vital insights into underlying brain activity through the examination of fluctuation and complexity patterns.

#### 3.4.2 Frequency domain features

Frequency domain features play a crucial role in the interpretation of EEG signals, since they are necessary for the diagnosis of neurological illnesses and for monitoring the brain's activity during the performance of cognitive functions. This research computed the subsequent features in the frequency domain:

- *Identification of central tendency features using EEG band's spectral features:* The spectral analysis of the four frequency bands (delta, theta, alpha, and beta) in an EEG signal can be utilized for determining central tendency attributes such as mean, median, mode, variance, standard deviation, kurtosis, skewness, minima, and maxima (Siddiqa et al., 2023). The central tendency of a dataset can be defined as the tendency of a dataset to accumulate around the average value or center of the dataset. A measure of the central tendency can offer insights into the common or predominant values found in a dataset. They have the ability to depict and provide a concise overview of data distributions. In order to compute central tendency characteristics based on spectral statistics, it was first necessary to determine the power spectral density (PSD) of the EEG data that was initially determined (Arif et al., 2023). Using Welch's method, PSD is calculated by segmenting the EEG signal into overlapping windows, computing the Fourier transform for each segment, and averaging the spectra to estimate the PSD. A more detailed spectral analysis of the EEG signal can be obtained by using this method. As a default, the resolution parameter is set to none. By doing this, the function determines the segment length automatically based on the input data length. As a default, the behavior attempts to strike a reasonable balance between frequency resolution and computational efficiency. Subsequently, the PSD has been subdivided into distinct frequency ranges: delta (0.5–3 Hz), theta (4–7 Hz), alpha (8–12 Hz), and beta (13–30 Hz). Afterwards, a total of 32 features representing central tendency of each frequency band were computed using frequency band spectral statistics.
- *Norm power of four EEG bands:* The normalized power is calculated by dividing the power inside each frequency band by the integral of the overall power spectral density (PSD) across all frequencies. By normalizing the power levels, it ensures a justifiable comparison of power levels across various frequency bands, while taking into consideration the fluctuations in the total power spectrum of the EEG signal. The normalized power values are useful parameters for classifying infant sleep stages because they represent the relative contribution and distribution of brain activity in specific frequency ranges.
- *Average frequency of four EEG bands:* The average frequency of each of four EEG band is determined by multiplying the frequencies within the relevant frequency indices by their respective PSD values. Subsequently, those values are added together, and the outcome is divided by the total sum of the PSD values within the specified frequency range. This calculation yields a weighted average frequency that signifies the central point or the most prominent frequency within the particular range of frequencies under consideration. This technique enables a numerical evaluation of the spectrum properties of the EEG data and offers a valuable understanding of the frequency distribution within each EEG band. It also aids in the classification of various sleep stages in neonates.
- *Maximum power of four EEG bands:* The maximum power of each EEG frequency band is determined by determining the frequency indices in the PSD that correspond to the specific frequency range of interest for each band. The indices are derived by comparing the frequency values with the lower and upper frequency limitations specified for each band. The highest PSD value within these specific frequency indices is subsequently obtained for each time point, resulting in the peak power level within the corresponding frequency range. In EEG signals, time points are discrete instances where the PSD can be estimated. This computation allows for the determination of the maximum intensity of brain activity within each distinct frequency band and offers vital insights into the prevailing power peaks found in the EEG signal.
- *EEG band ratios:* The power ratios between EEG frequency bands are calculated by dividing the normalized power of one band by the normalized power of another band. These ratios, such as the delta-theta ratio, alpha-beta ratio, delta-alpha ratio, theta-beta ratio, delta-beta ratio, and theta-alpha ratio, enable the evaluation of the proportional distribution of power and interactions among different frequency bands. The ratios are calculated using the normalized power values derived from the PSD analysis of the EEG data. The normalized power quantifies the relative impact of a particular frequency range in the complete power spectrum. The power ratios are obtained by dividing the normalized power of one band by the normalized power of another band. These ratios offer vital information into the equilibrium and supremacy of brain activity across various frequency ranges. Their contribution involves analyzing EEG data to characterize different sleep stages in newborns, providing insights on the relative importance of specific frequency components in the EEG spectrum.
- *Fast fourier transform (FFT):* By employing FFT, it is possible to examine the time-domain EEG signal by interpreting it into the frequency domain and analyzing its constituent frequency components. The input EEG data was subjected to a FFT to calculate its frequency spectrum. Subsequently, the 10 frequencies with the most significant FFT values were selected.

Consequently, all the above mentioned characteristics can be used to create automated sleep staging algorithms that have the potential to enhance the identification and treatment of infant's sleep disorders.

### 3.5 Feature importance and feature selection

In order to classify sleep states using EEG, we need to define what features in the frequency and time domains are the most informative. By using these techniques, we can distinguish sleep stages by using the most informative features. Using machine learning models, you can achieve better performance and more accurate results by selecting and emphasizing features (Ilyas et al., 2020). In this research, Principal Component Analysis (PCA) is utilized to select and prioritize features. The PCA algorithm determines which of the principal components captures the greatest proportion of variance in a dataset by analyzing its variance (Wold et al., 1987). The explained variance ratio can be used to determine a subset of principal components can be selected to reduce the dimensionality of the data. High variances indicate that the number of features in the dataset captures as much information as possible. By preserving the variance in the dataset, information which is the most important and relevant to the data can be preserved, and at the same time, the least important data can be eliminated. As a result of the designed PCA, 95% of the variance in the EEG was explained by the most informative features. A small number of principal components account for 95% of the variance in the dataset. After scaling the dataset and performing PCA, we found that a few principal components captured most of the variance. Using the columns that have been selected and the variable that is being targeted, a new dataframe is generated based on how many principal components there are. As a result, the information relevant to the prediction of the variable that is being targeted remains, and at the same time, the data is reduced in dimensionality. In the original dataset, 94 features from preprocessed EEG data were extracted. The resulting dataframe is used to classify sleep into five states using an LSTM model. However, based on PCA results, a total of 21 features have been decided upon for further consideration.

### 3.6 Synthetic minority oversampling technique analysis

SMOTE is a widely utilized data augmentation approach employed to deal with class imbalance in machine learning (Fernández et al., 2018). It is especially efficient when handling datasets in which one class is considerably less represented than the other. The process involves generating artificial data points for the underrepresented category by interpolating between adjacent examples. The objective of this strategy is to create more synthetic instances that closely resemble the existing samples from the minority class, hence enhancing their presence in the dataset (Fernández et al., 2018). The creation of synthetic samples includes the subsequent steps:

1. Determine the instances belonging to the minority class: Initially, the dataset is examined to identify the instances that belong to the minority class.
2. Randomly choose an instance *x*_*i*_ from the specified minority class instances.
3. Locate the k nearest neighbors: The k nearest neighbors of the given instance are determined using a selected distance metric, such as Euclidean distance (Li et al., 2024).
  (5) $$\begin{array}{l} \hat{x_{i}}=K_{i}x_{i} \end{array}$$
4. Choose one of the k nearest neighbors at random: A single neighbor is selected at random from the k nearest neighbors.
5. Create a synthetic instance *x*_*new*_: A novel synthetic instance is generated by interpolating between the selected instances and the chosen neighbor. This is achieved by employing a random selection process to choose a point located on the line segment that connects the two instances (Li et al., 2024).
  (6) $$\begin{array}{l} x_{new}=x_{i}+\left(\hat{x_{i}}-x_{i}\right)\delta \end{array}$$
  Interpolation between the *x*_*i*_ and $\hat{x_{i}}$ is controlled by δ, a value between 0 and 1. The value of δ specifies the extent of “smoothing” or “stretching.” The closer the synthetic samples are to the originals, the smaller the value of δ, and the farther they are from them, the larger the value.
6. Iterate the procedure: Steps 2 to 5 are iterated until the required extent of oversampling of the minority class is attained.

**Figure 3:**
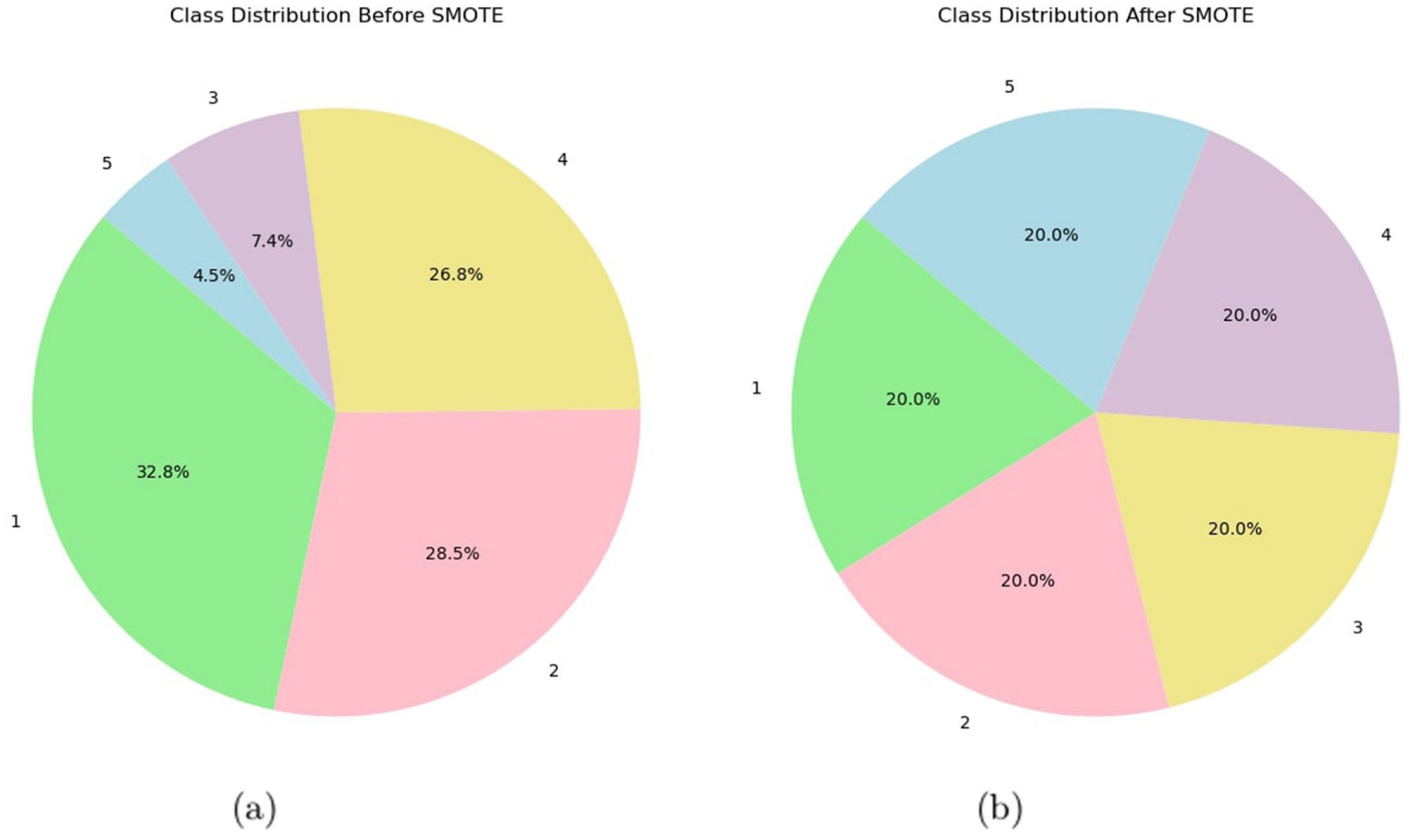
**(a)** Pie class distribution before SMOTE and **(b)** pie class distribution after SMOTE.

When applying SMOTE in the analysis of EEG features, the default delta value was used for oversampling, as specified by the SMOTE implementation. By defaulting the delta value, the implementation process becomes easier, ensuring a standard oversampling level without the need to tweak parameters manually, thereby making class imbalances easier to handle. The SMOTE algorithm is utilized in this specific study, employing the implementation provided by the scikit-learn module. The SMOTE function begins execution with a random state of 42. The effectiveness of the SMOTE technique is assessed by computing and presenting the counts of the resampled labels using a Pandas series. This analysis offers valuable information on the distribution of the balanced classes following the implementation of SMOTE. Figure 3 shows pie class distribution before and after SMOTE. This algorithm provides synthetic samples for the training set, improving the model's generalization and prediction capabilities (Gamel et al., 2024). A more precise representation of the fundamental distribution of the data is provided by this approach, which lessens the challenges faced by imbalanced datasets. The proposed methodology thus eliminates class imbalances and improves the performance of the model by training it on a more representative and balanced dataset. Using SMOTE, data leakage was prevented and model evaluation was ensured in this research after the train-test split. As a result of applying SMOTE only to the training set, the test data was kept intact, enabling us to assess model performance accurately. By doing so, the test set remains intact, simulating real-world conditions and enhancing model generalization.

### 3.7 Long short-term memory

An LSTM (Long Short-Term Memory) model is a variant of a recurrent neural network (RNN) that addresses long-term dependencies in sequential data. When processing long sequences, traditional RNNs struggle to capture information from earlier time steps due to the vanishing gradient problem. It can process entire sequences of data, not just individual data points, due to its feedback connections, unlike traditional neural networks. As a result, it is very effective at identifying and predicting patterns in sequential data, such as time series, text, and speech. As a powerful tool for artificial intelligence and deep learning, LSTMs are enabling breakthroughs in a wide range of fields by capturing valuable insights from sequential data.

#### 3.7.1 LSTM architecture

**Figure 4:**
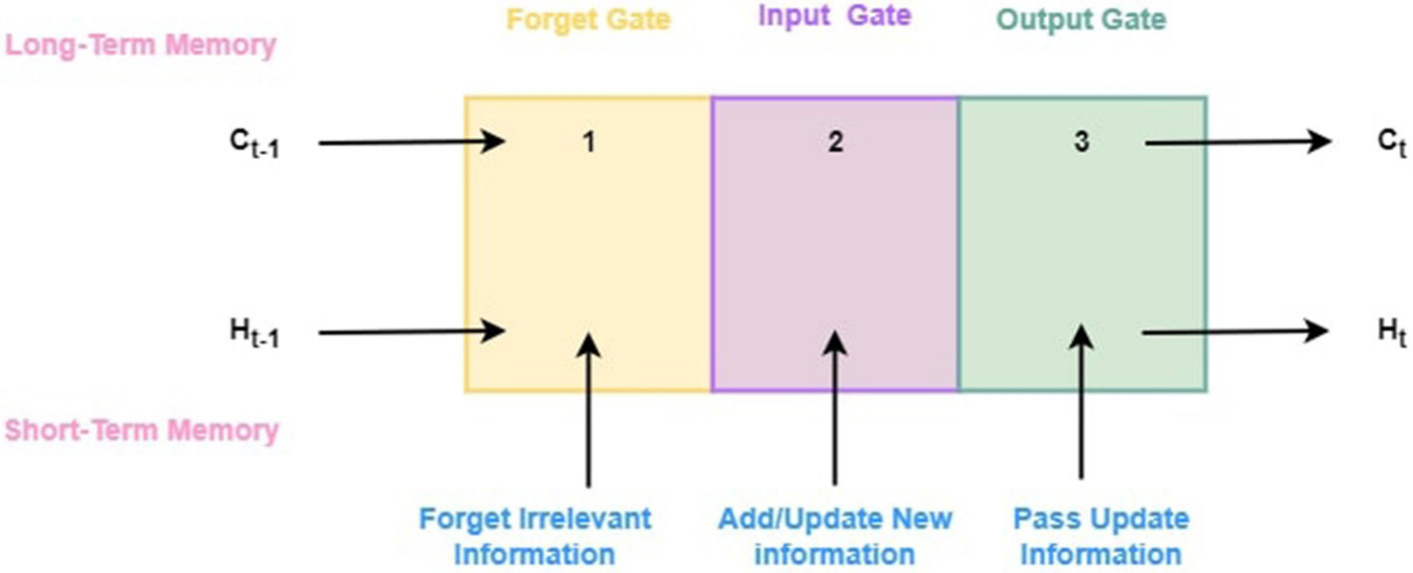
General architecture of LSTM model.

A LSTM network resolves the problem of vanishing gradients faced by RNN. At a high level, LSTM functions similarly to an RNN cell. Figure 4 illustrates its internal workings. As shown in Figure 4, the LSTM network architecture is composed of three components, each of which performs a specific task. Based on the previous timestamp, the first component determines whether the information is relevant or not. Using the input in this cell, the second component tries to learn new information. Finally, in the third component of the cell, the current timestamp is passed on to the next timestamp. The single-time step of the LSTM is considered to be one cycle. Gates are three components of LSTM units. The flow of information between the memory cell and the LSM cell is controlled by them. The forget gate is the first gate, the input gate is the second gate, and the output gate is the last gate. LSTM units composed of these gates and memory cells are similar to layers of neurons in traditional feed-forward neural networks, with each neuron having a current state and a hidden layer. Following is the step-by-step explanation of each gate:

1. **Forget gate:** This gate determines which information from the previous cell state should be discarded. Using the sigmoid activation function, which squashes values between zero and one, the forget gate output (*f*_*t*_) is calculated from the current input (*x*_*t*_) and the previous hidden state (*h*_*t*−1_).
  A forget gate can be described mathematically as follows:
  (7) $$\begin{array}{l} f_{t}=\sigma \left(W_{f}\cdot \left[h_{t-1},x_{t}\right]+b_{f}\right) \end{array}$$
  In this equation, σ represents sigmoid function, *W*_*f*_ represents the forget gate's weight matrix, [*h*_*t*−1_, *x*_*t*_] represents the concatenation of the previous hidden state with the current input, and *b*_*f*_ is the gate's bias term.
2. **Input gate:** As the input gate determines the amount of new information to be stored in the state of the cell, it takes into account both the current input and the previous hidden input (*h*_*t*−1_). A sigmoid activation function is used to compute the input gate output (*i*_*t*_).
  Input gates are mathematically defined as follows:
  (8) $$\begin{array}{l} i_{t}=\sigma \left(W_{i}\cdot \left[h_{t-1},x_{t}\right]+b_{i}\right) \end{array}$$
  (9) $$\begin{array}{l} C_{t}=\tanh\left(W_{C}\cdot \left[h_{t-1},x_{t}\right]+b_{C}\right) \end{array}$$
  In this case, *W*_*i*_ and *W*_*C*_ stands for the weight matrices associated with the input gate, *h*_*t*−1_ and *x*_*t*_ stand for the previous hidden state and current input, while *b*_*i*_ and *b*_*C*_ stands for the bias terms associated with the gate.
3. **Output gate:** By comparing the current input (*x*_*t*_) with the previous hidden state (*h*_*t*−1_), it determines which parts of the cell state should be output. The output gate output (*o*_*t*_) is determined by the sigmoid activation function.
  An output gate's mathematical equation is as follows:
  (10) $$\begin{array}{l} o_{t}=\sigma \left(W_{o}\cdot \left[h_{t-1},x_{t}\right]+b_{o}\right) \end{array}$$
  (11) $$\begin{array}{l} h_{t}=o_{t}\cdot tanh\left(C_{t}\right) \end{array}$$
  Here, *W*_*o*_ represents the weight matrix associated with the output gate, [*h*_*t*−1_, *x*_*t*_] represents the concatenation of the previous hidden state and the current input, while *b*_*o*_ represents the output gate bias term and *h*_*t*_ shows the output for hidden state.

In an LSTM cell, the gate outputs (*f*_*t*_, *i*_*t*_, *o*_*t*_) are important for controlling information flow. As a result, they determine which parts of the previous cell state should be forgotten, which new information should be added to the cell state, and which parts of the updated cell state should be hidden.

### 3.8 Proposed model architecture

**Figure 5:**
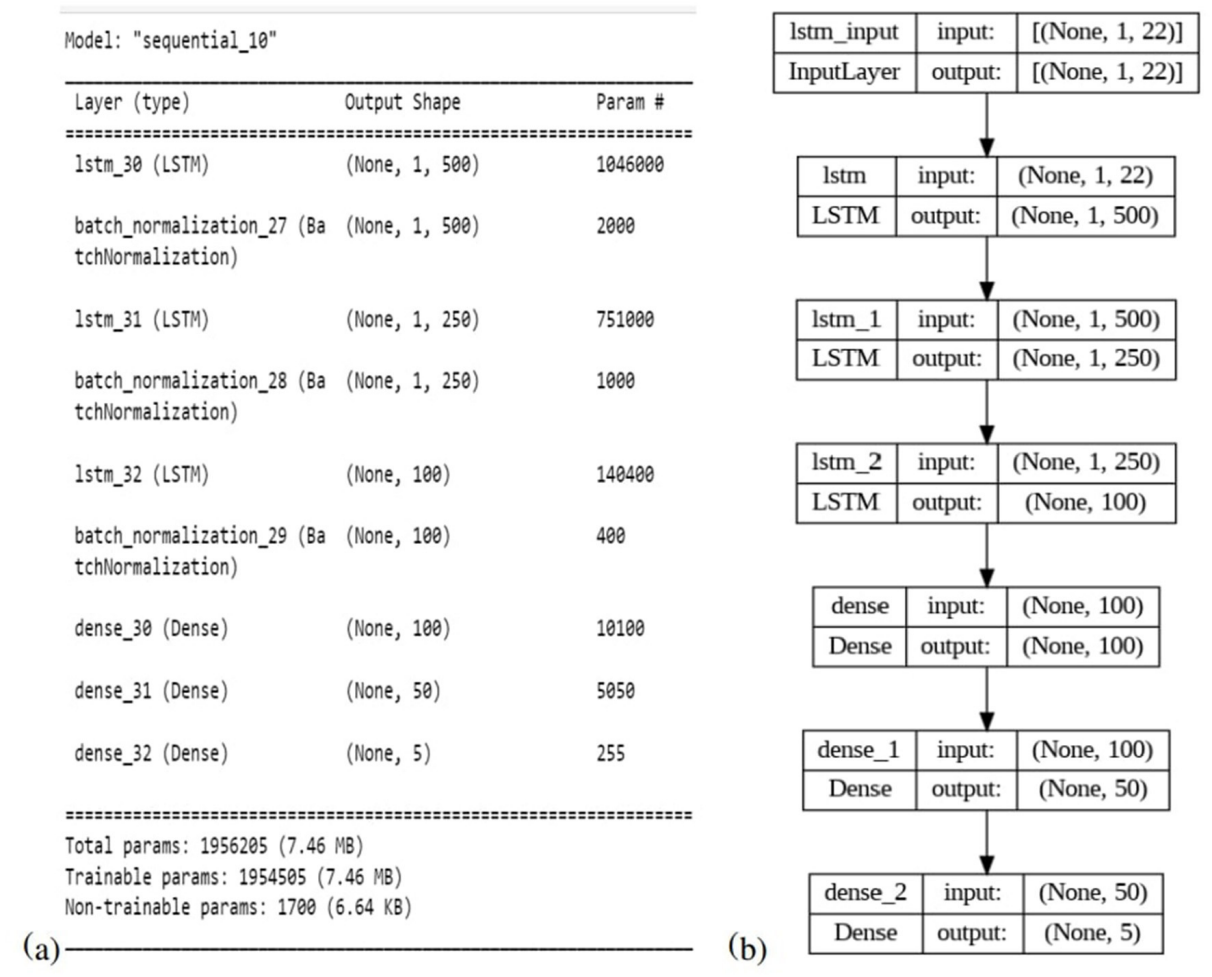
**(a)** Detailed information about LSTM layers. **(b)** An overview of the model's architecture.

The proposed LSTM model for neonatal sleep staging is presented in this subsection with detailed descriptions of the mathematical model, its architecture, and all parameters. In this paper, an eight-layer LSTM architecture has been proposed in order to represent the LSTM. Figure 5 provides a comprehensive depiction of the model's structure and offers in-depth insights into its individual layers. Sequentially stacking LSTM layers, this model consists of three layers with different regularization levels and units. There are 500 units in the first layer, and it returns sequences, while there are 250 units in the second layer, and it also returns sequences. LSTM layers are regularized using L2 regularization with a factor of 0.0001 to prevent overfitting. The third layer does not return sequences and has 100 units. Each LSTM layer is followed by a batch normalization layer for speed and stability. After two dense layers of 100 and 50 units, respectively, and ReLU activation, a final dense layer with a number of units corresponding to the classification task's classes is added, and class probability is output using softmax activation. Adam's optimizer, cross-entropy loss function, and accuracy metric are used to compile the model. During training, the model's states and parameters are reset, and with a batch size of 128 and an early stopping with a patience of 10 is implemented. Table 2 presents details about all other hyper-parameters used in proposed LSTM. Experimentation was conducted in order to select and tune all hyperparameters in order to optimize performance and convergence during training. The model is trained and evaluated for one epoch using the data provided. Then the model's performance on the validation set is evaluated after each epoch.

**Table 2:** Details about hyper-parameters.

**Parameter**	**Value**
Epochs	50
Batch size	128
Optimizer	Adam
Kernel regularization	L2
Learning rate	1 × 10^−4^
Cross-validation k-folds	10
Loss function	Binary cross-entropy

### 3.9 Performance assessment metrics

In order to test and evaluate the proposed scheme, different performance metrics are used, including confusion matrix, accuracy, Cohen's kappa, recall, precision, Mathew's co-relation coefficient, and F1-score. In this study, the classification model is examined based on these metrics to determine whether it can accurately identify EEG patterns.

- *Confusion matrix:* An analysis of a classification model's quality is conducted using a confusion matrix. In multi-class classification, confusion matrixes show the number of correct and incorrect predictions for each class as a tabular representation of the model's performance. Identifying specific types of classification errors helps to improve the model's accuracy for individual classes. It is possible to evaluate the model's performance across multiple classes by calculating metrics such as precision, recall, and F1-score.
- *Accuracy:* The accuracy of machine learning (ML) algorithms is commonly measured as a percentage of correctly classified measurements. The formula (Ali et al., 2020) can be used to calculate this percentage:
  (12) $$\begin{array}{l} Acc=\frac{\left(TP+TN\right)}{\left(TP+TN+FP+FN\right)} \end{array}$$
- *Cohen's Kappa:* The Cohen's Kappa is commonly used to estimate how well two raters agree. It is also used to determine the performance of classifiers. The confusion matrix cells are used to calculate it as follows (Chicco et al., 2021):
  (13) $$\begin{array}{l} kappa=\frac{2\left(TP\cdot TN-FP\cdot FN\right)}{\left(TP+FP\right)\cdot \left(FP+TN\right)+\left(TP+FN\right)\cdot \left(FN+TN\right)} \end{array}$$
  When Kappa is –1, it is the worst, and when it is +1, it is the best.
- *Recall:* Recall in machine learning refers to how well an algorithm can identify a class based on a set of sampled data. In mathematics, recall is expressed as Shaukat et al. (2020):
  (14) $$\begin{array}{l} Rec=\frac{TP}{TP+FN} \end{array}$$
- *Precision:* In order to determine a model's precision, it must be able to identify a significant number of relevant items. Accordingly, it can be written as follows (Shaukat et al., 2020):
  (15) $$\begin{array}{l} Pre=\frac{TP}{TP+FP} \end{array}$$
- *Matthews correlation coefficient (MCC):* MCC measures the difference between the predicted values and recorded values. The confusion matrix is used to calculate this (Chicco et al., 2021):
  (16) $$\begin{array}{l} MCC=\frac{TP\cdot TN-FP\cdot FN}{\sqrt{\left(TP+FP\right)\cdot \left(TP+FN\right)\cdot \left(TN+FP\right)\cdot \left(TN+FN\right)}} \end{array}$$
  MCC value of –1 is the worst, while a value of +1 is the best.
- *F1-Score:* F1-score is the combination of recall and precision, making it a powerful metric. It is mathematically computed by Shaukat et al. (2020), and Bing et al. (2022):
  (17) $$\begin{array}{l} F1\text{\_}Score=\frac{2\times Pre\times Rec}{Pre+Rec} \end{array}$$
- *Accuracy line graph:* The accuracy line graph permits comparisons, thresholds, and determinations of the model's performance over a range of values. This graph displays accuracy values along the Y-axis and fold counts along the X-axis. On the graph, every data point represents an individual cross-fold's accuracy. As the number of folds increases, the line connecting the data points indicates a trend in accuracy.
- *Validation accuracy curve:* Validation accuracy curves for N-fold cross-validation show how accuracy changes over time for each of the N folds. One can visualize the model's performance across different subsets of data by plotting validation accuracy vs. training iterations or epochs. As well as providing valuable insights into the model's learning behavior, this visualization allows assessment of the model's stability and generalization ability.

## 4 Results

**Figure 6:**
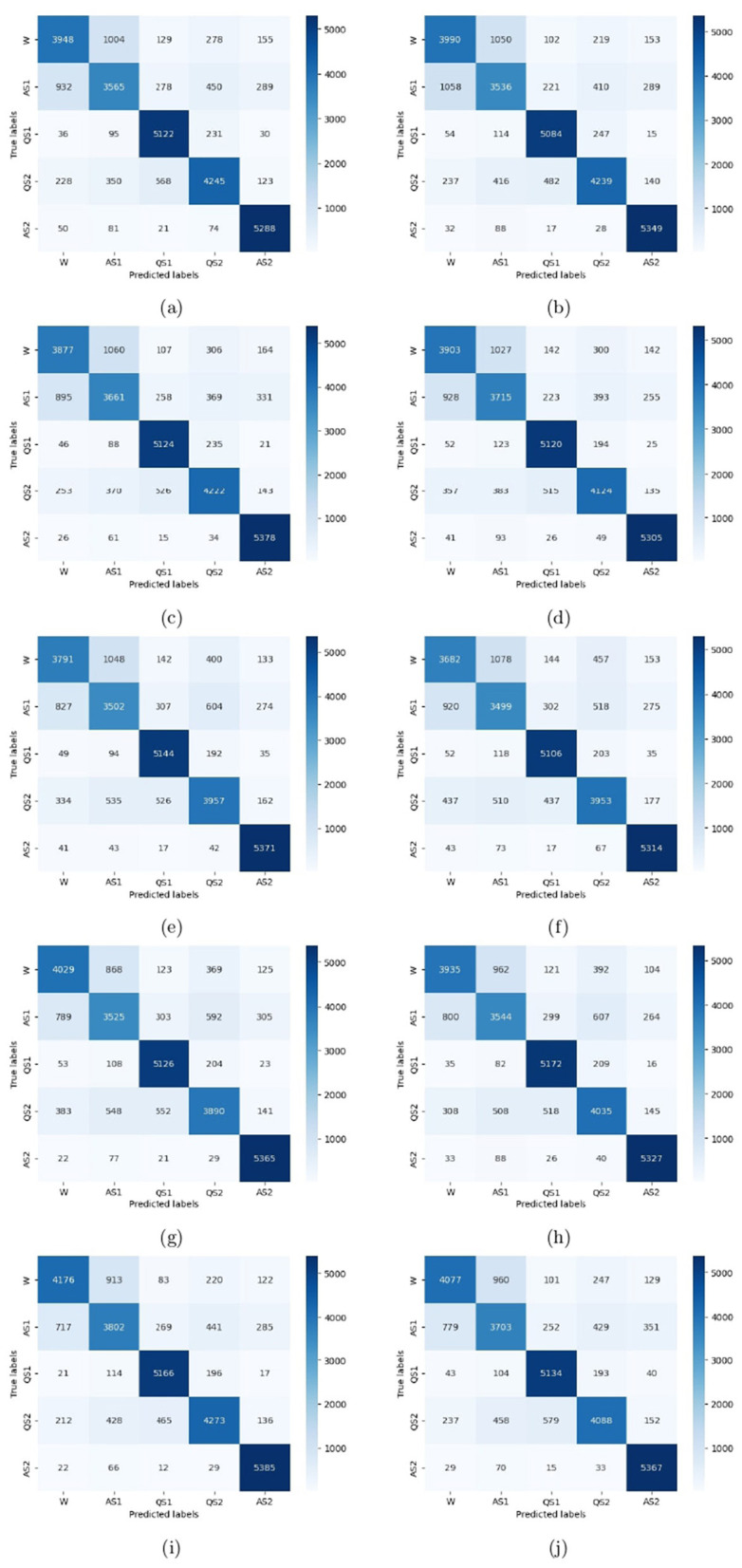
Confusion matrices for the channels: **(a)** F3, **(b)** F4, **(c)** C3, **(d)** C4, **(e)** P3, **(f)** P4, **(g)** T3, **(h)** T4, **(i)** Left side, and **(j)** Right side.

**Figure 7:**
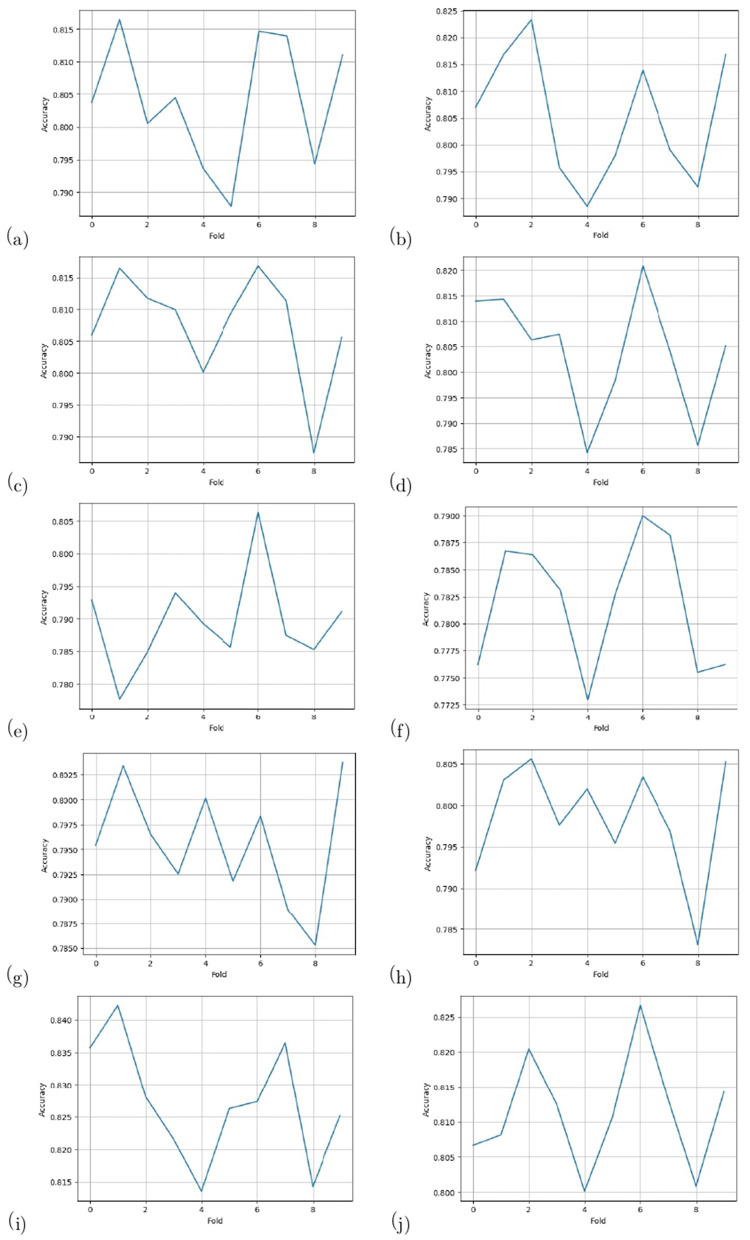
Accuracy line graphs for the channels: **(a)** F3, **(b)** F4, **(c)** C3, **(d)** C4, **(e)** P3, **(f)** P4, **(g)** T3, **(h)** T4, **(i)** Left side, and **(j)** Right side.

**Figure 8:**
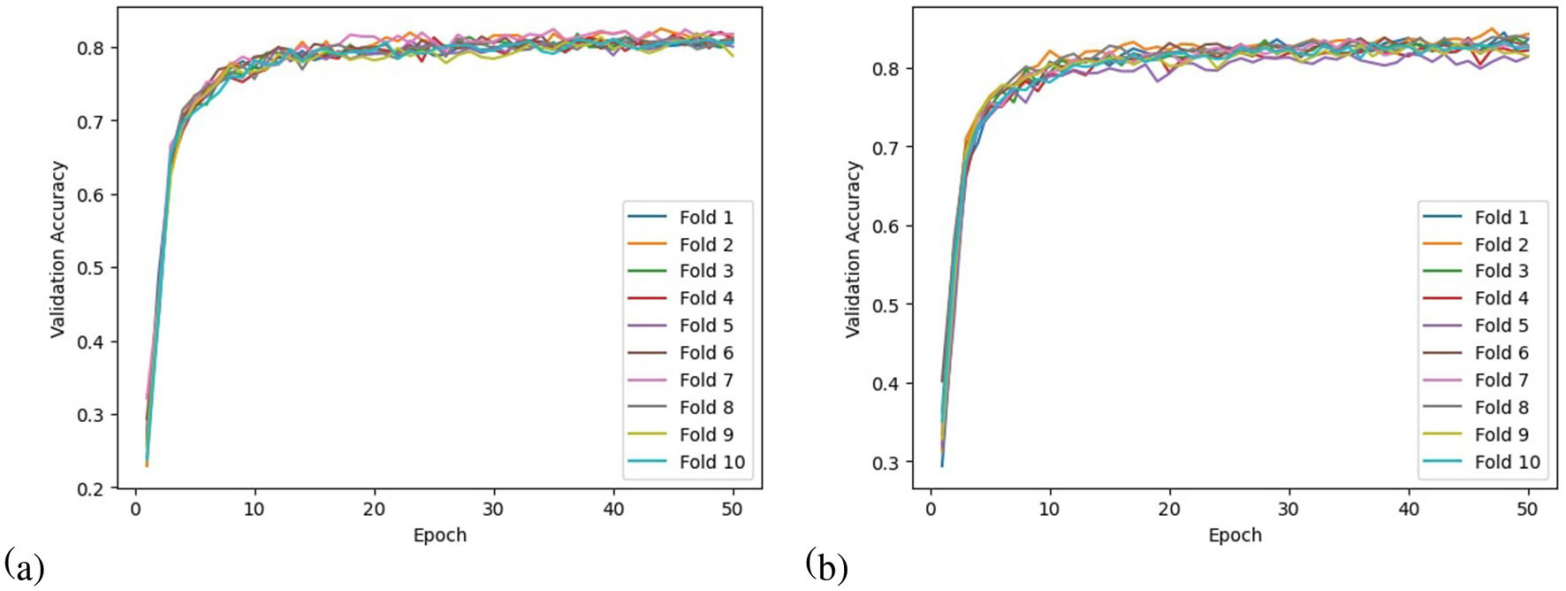
Validation accuracy curves for the channels: **(a)** C3 and **(b)** Left side.

To evaluate the performance of the model, a 10-fold cross-validation procedure was used. The data sets were shuffled randomly beforehand to avoid bias. Ten subsets of data were used for this methodology, with one set serving as the testing set and the remaining nine sets serving as the training set. Thus, it was possible to assess the generalization performance of the model in a way that minimized the leakage between the training and testing phases. As a result of the rigorous methodology used in this study, the performance of the proposed model has been rigorously and unbiasedly evaluated. In this study, the F3-channel and C3-channel show the greatest confusion matrix values when it comes to single-channel EEG data. In Figure 6, confusion matrices for the combinations of channels on the left and right sides and all single channels are shown. Tables 3, 4 present the analytically computed values for each performance assessment metric. For the combinations of channels on the left and right sides and all single channels, a line graph showing the level of accuracy can be seen in Figure 7. The accuracy values are displayed on the Y-axis in Figure 7. In Figure 7, accuracy line graphs represent model performance during 10 cross-folds. Lastly, Figure 8 illustrates validation accuracy curves for C3 single-channel and a combination of four left-side channels.

**Table 3:** Single-channel EEG classification results for five states.

**Channel**	**Acc (%)**	**Kappa**	**Rec (%)**	**Pre (%)**	**MCC (%)**	**F1_Sco (%)**
F3	80.41 ± 0.94	0.76	80.41 ± 0.94	80.08 ± 1.06	76 ± 0.01	80.01 ± 1.02
F4	80.52 ± 1.14	0.76	80.52 ± 1.14	80.20 ± 1.20	76 ± 0.01	80.24 ± 1.21
C3	80.75 ± 0.82	0.76	80.75 ± 0.82	80.39 ± 0.91	76 ± 0.01	80.41 ± 0.89
C4	80.40 ± 1.13	0.76	80.40 ± 1.12	80.17 ± 1.17	76 ± 0.01	80.15 ± 1.15
P3	78.94 ± 0.72	0.74	78.94 ± 0.72	78.53 ± 0.78	74 ± 0.01	78.54 ± 0.76
P4	78.18 ± 0.58	0.73	78.18 ± 0.58	77.72 ± 0.59	73 ± 0.01	77.83 ± 0.58
T3	79.56 ± 0.58	0.74	79.56 ± 0.57	79.16 ± 0.72	75 ± 0.01	79.18 ± 0.64
T4	79.84 ± 0.67	0.75	79.84 ± 0.67	79.46 ± 0.71	75 ± 0.01	79.51 ± 0.71

**Table 4:** Four-channel EEG classification results for five states.

**Channel**	**Acc (%)**	**Kappa**	**Rec (%)**	**Pre (%)**	**MCC (%)**	**F1_Sco (%)**
Four-channel (Left)	82.71 ± 0.88	0.78	82.71 ± 0.88	82.47 ± 0.94	78 ± 0.01	82.46 ± 0.92
Four-channel (Right)	81.14 ± 0.77	0.76	81.14 ± 0.77	80.87 ± 0.83	77 ± 0.01	80.83 ± 0.85

Left side channels: F3, C3, P3, T3 & Right side channels: F4, C4, P4, T4.

## 5 Discussion

Using an LSTM classifier, this study proposes a method of neonatal sleep staging based on single-channel and then four-channel EEG data. In order to determine which EEG channel is important in neonatal sleep staging and which channels are most appropriate for five-state classification, single-channel EEG data needs to be used to determine which channel and which side of the head should be used. After preprocessing the EEG data collected from 64 infants, 16,803 segments are left for testing and training of channels F3, F4, C3, C4, P3, P4, T3, and T4. EEG data is then processed for 94 linear and non-linear features. These features are divided into the time and frequency domains. A total of 27 statistical parameters were included in the analysis for the time domain, including mean, median, standard deviation, minima, maxima, range, skewness, and kurtosis. The data was further processed to extract nonlinear features such as Detrended Fluctuation Analysis (DFA), Lyapunov exponents, and Multiscale Fluctuation Entropy. A FFT is used in order to extract frequency domain features by separating ten features based on their FFT values and then using spectral statistics to calculate 36 central tendency features for each frequency band in the first place. Through the capture of complex dynamics and irregularities in neonatal EEG signals, these features allow a better understanding of neonatal sleep patterns. By preserving 95% of the variance of the data, we reduced the dimensionality and retained the most informative features by applying Principal Component Analysis (PCA). The Synthetic Minority Oversampling Technique (SMOTE) is also applied for data augmentation to address the imbalanced nature of the dataset. By using this technique, we were able to improve the classification model by balancing the classes.

A description of the proposed LSTM has already been provided in Section 3. A model is used to classify sleep states using 94 features that are obtained from each channel of the EEG signals. Four channels on the left side and four channels on the right side are combined in order to determine the neonate's sleep states. Figure 5 shows the proposed LSTM in its entirety. The description of all layers and their types, as well as their parameters, can also be found in Figure 5. It has been tried many times to get the best performance from the model by testing kernel regularization, unit number, and activation function in the real world. A final choice was made by considering how to balance the complexity of the model with the generalizability of the models after testing a variety of combinations and assessing the effectiveness of each combination. The performance evaluation step involved a 10-fold cross-validation procedure. This methodology used ten subsets of data, nine of them as training sets and one as a test set. In order to eliminate bias in the data sets, the data sets were shuffled prior to the analysis at random. Thus, the generalization performance of the proposed model could be assessed without leaking information between the phases of training and testing of the model. This unbiased evaluation method was used to rigorously and unbiasedly evaluate the performance of the proposed method. In this study, accuracy and other matrices values are expressed as Mean ± SD. Using the mean, one can see how accurate the experiments are, while the standard deviation indicates how uncertain or variable the accuracy measurements are. Averaging the individual accuracy values obtained from multiple trials yielded the mean accuracy, whereas the standard deviation measures how far the accuracy measurements are from the mean. By presenting the accuracy results in this way, we can gain insight into both their central tendency and their variability. In Tables 3, 4, data from single channel and four channel EEGs for five-state neonate sleep classification is used. In single-channel five-state classification, the F3, F4, C3, and C4 channels achieve maximum mean accuracy and kappa. For the F3 channel, the accuracy and the kappa values are 80.41 ± 0.94% and 76%, respectively. For the F4, these values are 80.52 ±1.14 % and 76%. For the C3 channel, these values are 80.75 ± 0.82 and 76%, respectively. For the C4, these values are 80.40 ± 1.13 and 76%, respectively. There is also evidence to suggest that by combining four left-side channels (F3, C3, P3, and T3), the highest mean accuracy and kappa values can be achieved, with accuracy and kappa values of 82.71 ± 0.88 and 78%, respectively. Right side electrode combinations (F4, C4, P4, and T4) have values of 81.14 ± 0.77 and 76%, respectively. In addition, accuracy line curves and confusion matrices for five states are also shown in Figure 7 in order to visualize the model's performance and learning progress. As shown in the above Table 3, for the classification of the five-state sleep stage of newborns, channels P3, P4, T3, and T4 are far less helpful than channels P3 and P4 in determining the sleep stage. However, F3, F4, C3, and C4 perform well. When there are four channels, left-side channels perform better than right-side channels. Even with fewer channels, performance is still favorable when the parameters relating to performance are compared with those presented in Tables 3, 4. It has been shown that sleep analysis can enhance the care of neonates and enable them to be monitored effectively in order to detect sleep-related abnormalities, such as sleep disorders, early in order to treat them early.

Comparisons of existing and proposed methods are presented in Table 5. This article and Zhu et al. (2023) refer to the same dataset, ensuring consistency and comparability in evaluating the models listed in Table 5. Most of the models in this Table have been evaluated on this dataset by Zhu et al. (2023), and the results obtained are also reflected in that Table. The proposed study uses datasets that are several times larger than those used in Ansari et al. (2020) and Ansari et al. (2018). On the basis of this dataset, these models were found to be underfitting. For adult sleep, Supratak et al. (2017) and Eldele et al. (2021) are presented. Taking into account the difference in sleep patterns between infants and adults, these models are prone to convergence problems and overfitting. Therefore, it is hard to transfer an adult sleep staging model directly to neonate data because this causes convergence problems and overfitting. The model needs to be modified to reflect the neonate's sleep characteristics. A serial recurrent neural network (RNN) is used as part of the TIL module in the model architecture in Zhu et al. (2023), which results in a lengthy training time and inefficient training.

**Table 5:** Comparison of existing and proposed methods.

**References**	**Algorithms**	**No. of channels**	**Accuracy**	**Kappa**
Ansari et al. (2020)	Conv-2d	8	52.3%	0.41
Ansari et al. (2018)	Conv-2d	8	53.5%	0.48
Zhu et al. (2023)	MS-HNN	1	75.4%	0.72
Zhu et al. (2023)	MS-CNN	1	69.3%	0.65
Supratak et al. (2017)	DeepSleepNet	2	69.8%	0.64
Eldele et al. (2021)	AttnSleep	1	68.0%	0.65
Siddiqa et al. (2024)	Multi-Branch CNN	1	74.27%	0.61
Siddiqa et al. (2024)	Multi-Branch CNN	4	75.36%	0.63
This study	LSTM	1	80.75% ± 0.82%	0.76
This study	LSTM	4	82.71% ± 0.88%	0.78

Based on the experiments, limitations and future directions should be identified. Using only EEG signals as inputs to the proposed scheme in this paper is the primary objective of this paper, which is to assess its feasibility and reliability. In this study, electrooculography (EOG), electromyography (EMG), and electrocardiography (ECG) were not used. However, they could be used in the future to assess neonatal sleep with various input signals. Further improvement could be accomplished by using Transformer (Vaswani et al., 2017) rather than CNN to learn. Additionally, all subjects were randomly divided into a set of training subjects and a set of test subjects in this study. Future research can increase the accuracy of the classification of neonatal sleep stages by incorporating an independent set of subjects in the training and testing phases. As a result, the performance of MFE in the context of sleep staging should be compared to Multiscale Dispersion Entropy and Multiscale Fluctuation Dispersion Entropy. A number of studies have shown that these methods are better at detecting meaningful patterns (Zandbagleh et al., 2023; Chakraborty et al., 2021). In addition to potential overfitting from the Multi-Branch CNN, its limited capacity for hierarchical temporal learning may have made it difficult to capture long-range EEG signal dependencies. Further, its inefficiency in learning sequential patterns and its sensitivity to signal variability could have adversely impacted generalization and contextual understanding. In comparison to 1D CNNs, LSTM models generally perform better when dealing with time series data. LSTM networks, on the other hand, yield more accurate results by retaining long-term dependencies, interpreting context over sequences, and capturing fine-scale changes in EEG data, making them more suitable for effectively identifying five distinct sleep states. With the integration and evaluation of these techniques, future research can enhance sleep staging algorithms.

## 6 Conclusion

Using an LSTM classifier that takes into account features in the time and frequency domains, this study proposes an efficient and accurate classification of neonatal sleep states based on EEG, using single and multi-channel EEG data. A combination of Detrended Fluctuation Analysis (DFA), Multiscale Fluctuation Entropy, and Lyapunov Exponents is used to analyze the data in this study. PCA is used to select features. With the use of both single-channel as well as multiple-channel EEG data, it achieves favorable and comparable results. The number and placement of channels play a critical role in the optimal electrode configuration for the assessment of neonatal sleep stages and the most effective channels in five states. Using a variety of electrode setups, the purpose of this study was to evaluate the accuracy of sleep stage classification for neonatal sleep studies in order to reduce complexity and cost. The frontal and central EEG channels worked better independently or jointly, based on the results. In the future, neonate sleep staging can be simplified, comfort levels can be increased, and data analysis can be sped up by reducing the number of channels. Through sleep analysis, it is possible to detect sleep-related abnormalities, such as sleep disorders, early, allowing for more effective neonate care and monitoring of sleep. Also, the experimental results suggest that the proposed approach captures information effectively within a single channel, reducing computing load by reducing the number of channels, while maintaining good performance. Furthermore, including linear and non-linear features in the time and frequency domains of neonatal sleep staging can improve accuracy and provide insights into newborn sleep dynamics and irregularities.

## Data Availability

The datasets presented in this article are not readily available because due to privacy concerns. Requests to access the datasets should be directed to hafizaasha@yahoo.com.
